# Editorial: Trace element chemistry and health

**DOI:** 10.3389/fnut.2022.1034577

**Published:** 2022-10-18

**Authors:** Xinyu Wang, Yahao Zhao, Xueming Wu, Leqi Cui, Shuai Mao

**Affiliations:** ^1^Department of Medicinal Chemistry, School of Pharmacy, Xi'an Jiaotong University, Xi'an, China; ^2^Zhejiang Huayu Food Co., Ltd., Lishui, China; ^3^Department of Nutrition and Integrative Physiology, Florida State University, Tallahassee, FL, United States

**Keywords:** trace element, disease, analytical methods, pathogenesis, healthy

There are about 70 trace elements in human body that account for 0.005–0.01 wt % of body weight (1). Some trace elements are essential components of large molecules that have biological activities; therefore small variations in the levels of trace elements could significantly affect the activities of the relevant molecules (Figure 1). For example, as cofactors, copper, zinc, iron are essential in maintaining the activities of oxido-redox enzymes (2). Copper is an important component of ceruloplasmin and copper-zinc superoxide dismutase, and it can affect the activities of antioxidant enzymes such as catalase and glutathione peroxidase, and is closely related to the antioxidant process of the body. Trace elements could also negatively impact on human health (3). For example, imbalanced serum Zn and Cd can interfere with glucose levels, potentially leading to cardiovascular diseases. Elements such as As, Pb, Cd and Cu have also been associated with increased risks of cardiovascular disease and coronary heart disease. One indicator for assessing nutritional status and diagnosing malnutrition caused by elemental imbalance could be serum albumin level as epidemiological studies have found a relationship between its level and trace element concentrations. Long-term being exposed to hexavalent chromium may reduce serum albumin levels, and the research by Li et al. showed that albumin level had negatively correlated with the concentrations of Co, Cr, Cu, Fe, Mn, Se, Zn, Pd and other elements.

**Figure 1:**
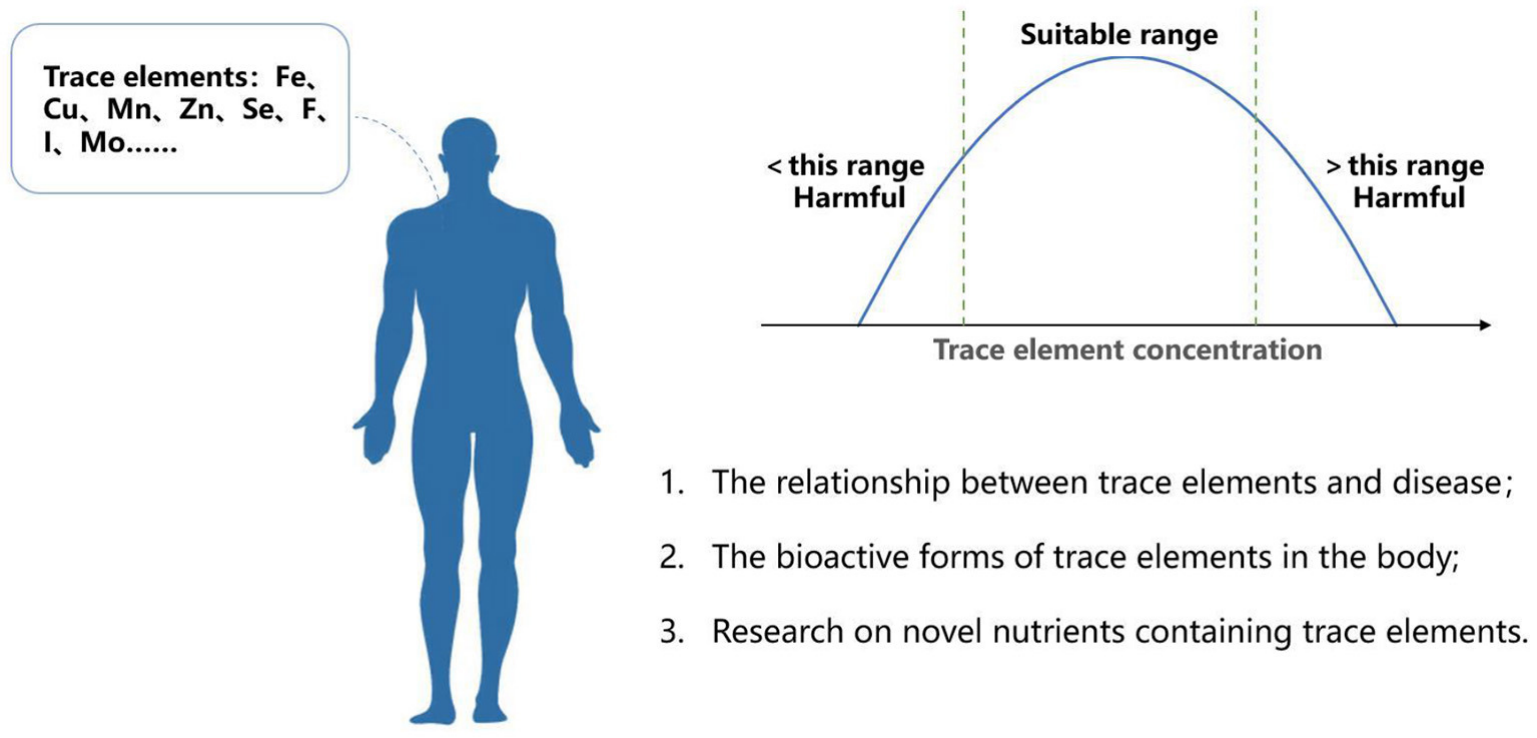
Trace elements in human nutrition and health.

The various elements in the body do not work independently and can therefore have antagonistically or synergistically effects (4, 5). Iron and manganese can interfere with each other's absorption in the digestive tract, while they also have the synergistic effect on hemogenesis. Appropriate dose of Se can mitigate the inhibition effects of as on antioxidants. And an animal study showed that the high selenium diet increased the excretion of arsenic in urine and feces, reduced the residual arsenic in kidney, improved the antioxidant level and reversed the immunosuppression caused by arsenic (Bai et al.) (6).

Both insufficient and excessive intake of trace elements could result in diseases. For example, Chinese adults' safe intake of iron is 12–42 mg/d for men and 20–42 mg/d for women. While iron deficiency leads to anemia, excessive iron in the body causes damages to heart, liver and pancreas (7, 8). Similarly, long term insufficient intake of zinc in adults (below 12.5 mg/d for adult men and 7.5 mg/d for adult women) can lead to decreased immunity, decreased digestive function and other symptoms. For infants and young children, it will compromise the cell growth, division and differentiation (5). While excessive zinc (above 40 mg/d) is associated with acute renal failure, atherosclerosis, hypertension, and coronary heart disease. There is a close relationship between selenium deficiency and Kaschin-beck disease (KBD). Se could improve cell aging and strengthen lipid peroxidation, which played a key role in articular cartilage damage in patients with KBD (9). Kang et al. found that the selenium levels of all subjects in Kaschin-Beck disease area were lower than normal after stopping the selenium supplementation. And they also found that excessive fluorine significantly inhibited the hydrolysis of T-2 toxin by inhibiting the expression of CES1 gene and protein and their hydrolytic activity, thus affecting patients with KBD (Jia et al.).

In addition to the amount of intake, the chemical state of trace elements also matters in terms of their toxicity and bioavailability. The existing state of elements can be divided into inorganic, organic or biological state (10). Inappropriate supplementation of trace elements (such as the use of inorganic salts) and environmental pollution cause elements to accumlate in the body as ions, which could adversely affect human health (5). Excessive Ni^2+^ causes damage to kidney; Cu^2+^ causes damage to the gastrointestinal tract; Pb^2+^ causes damage to the hematopoietic system and so on. Organic trace elements are those that combine trace elements with ligands (such as amino acids, protein, polysaccharides, and the like) in the form of salts, complexes, or chelates, such as Metal-MHA chelate, Selenium yeast, metal polysaccharide complex, Zn or Cr propionate and so on. While the biological state can only be synthesized through biotransformation and can be directly absorbed and utilized, with the highest bioavailability. Foods with higher contents of trace elements are currently a better utilization mode of biological trace elements. Comparing to inorganic state, trace elements in their organic or biological state are more readily absorbed by human body with less side effects, presumably because these are closer to their natural structures (5). For example, zinc gluconate, an organic zinc drug, has more curative effect and less side effect than the inorganic zinc drug (e.g., zinc sulfate). For the same reason, the greater toxicity of inorganic salt forms of selenium and chromium can be reduced by converting them into selenium yeast or chromium yeast (11).

In addition to understand the effects of elements on human health, how to develop analytical methods, basing on element chemistry, to monitor the elements concentration in the environment and in human body is equally important. Leng et al. synthesized a novel N_4_-based fluorescent probe that responds quickly, selectively and sensitively to Cu^2+^ in water. The probe has low cytotoxicity and good biocompatibility. Moreover, although it is already known that long term excessive or insufficient intake of trace elements leads to physiological abnormalities or diseases, it still remains unclear on the relationship between many elements and disease pathogenesis. Lastly, it is also crucial to better understand how to scientifically get trace element intake from diet and/or supplements. For the healthy development of mankind, these are the problems we need to face and solve in the future.

## Author contributions

All authors listed have made a substantial, direct, and intellectual contribution to the work and approved it for publication.

## Funding

This work was supported by the National Natural Science Foundation of China (21602169 and 81803490), and Xi'an Jiaotong University (334100038).

## Conflict of interest

Author XW was employed by Zhejiang Huayu Food Co., Ltd. The remaining authors declare that the research was conducted in the absence of any commercial or financial relationships that could be construed as a potential conflict of interest.

## Publisher's note

All claims expressed in this article are solely those of the authors and do not necessarily represent those of their affiliated organizations, or those of the publisher, the editors and the reviewers. Any product that may be evaluated in this article, or claim that may be made by its manufacturer, is not guaranteed or endorsed by the publisher.
